# Intestinal Radiation-Induced Stricture Favours Small Bowel Obstruction by Phytobezoar: Report of a Case

**DOI:** 10.1155/2009/482039

**Published:** 2009-06-30

**Authors:** Alessandra Quercioli, Franco Dallegri, Luciano Ottonello, Fabrizio Montecucco, Giacomo Borgonovo

**Affiliations:** ^1^First Clinic of Internal Medicine, Internal Medicine Department, University of Genoa, 16143 Genoa, Italy; ^2^Division of Cardiology, Geneva University Hospital, Faculty of Medicine, Foundation for Medical Researches, 1211 Geneva, Switzerland; ^3^Department of Surgical and Morphological Disciplines and Integrated Methodologies, University of Genoa, 16143 Genoa, Italy

## Abstract

Bezoars represent the fifth most frequent cause of acute small bowel obstruction. Phytobezoar is the most common type of bezoar. It is a concretion of undigestible fibers derived from ingested vegetables and fruits. We report a case of a woman with a 1-year history of recurrent epigastric and periumbilical abdominal pain with intermittent vomiting caused by phytobezoar of the terminal ileum. After careful investigation of the case and review of literature, we identified the factor involved in bezoar formation as radiation-induced ileal stenosis due to previous treatment for a pelvic tumour. This report provides evidence to consider phytobezoar as a possible cause of small bowel obstruction in patients previously treated with abdominal radiotherapy.

## 1. Introduction

Bezoars are composed by ingested foreign materials that accumulate within the gastrointestinal tract [1]. A phytobezoar is a particular kind of bezoar composed of indigestible cellulose, tannin, and lignin derived from ingested vegetables and fruits (especially persimmons) [2]. Phytobezoar represents a rare cause of intestinal obstruction that must be considered in the diagnostic workup and treated appropriately. To our knowledge, this is a rare case report of small bowel obstruction due to a phytobezoar of the ileum, secondary to radiation-induced terminal ileal stricture.

## 2. Case Report

**Figure 1:**
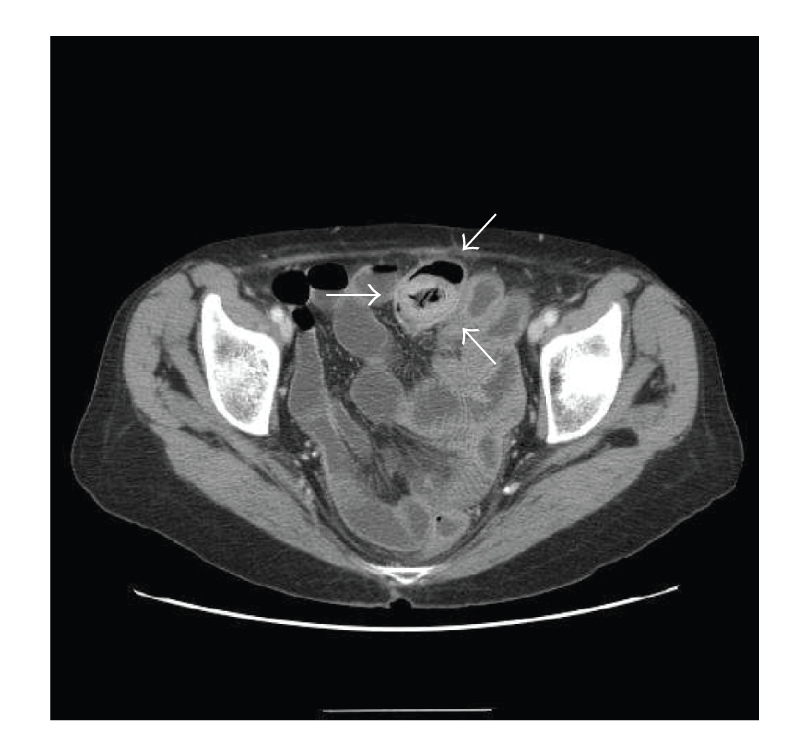
Axial abdominal CT scan showed an ovoid, nonhomogeneous, intraluminal filling defect about 3 cm in size (arrows). CT demonstrates no dilated small bowel loops, and the intraluminal mass with air retained in its interstices, which correspond to the phytobezoar.

**Figure 2:**
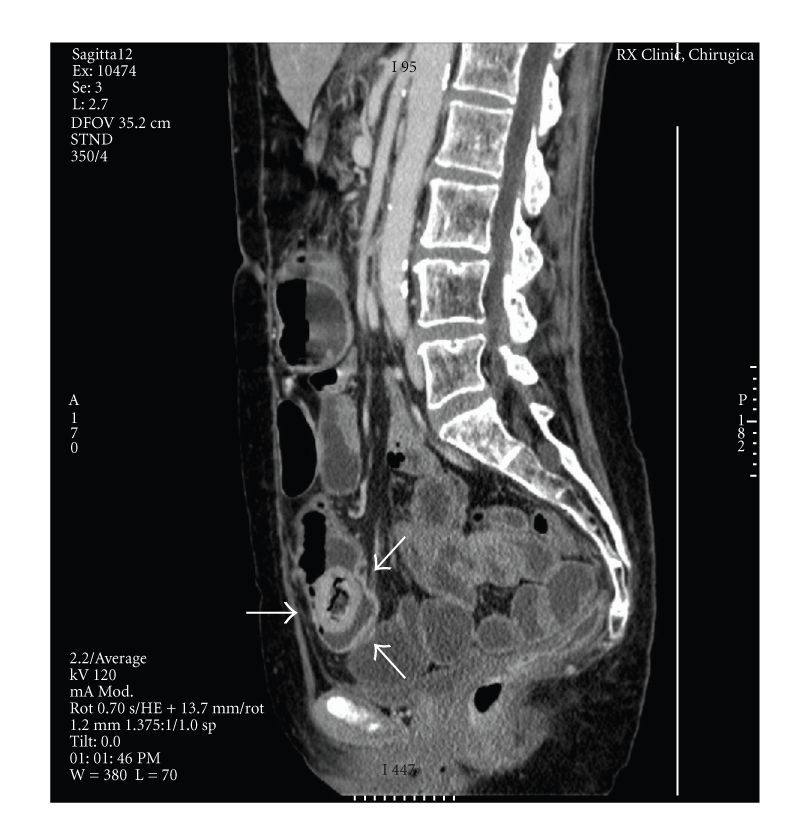
Sagittal contrast-enhanced scan shows loops of small bowel containing air and fluid levels suggestive of obstruction and an intraluminal mass with mottled appearance characteristic of bezoar (arrows) confined inside the small bowel at the pelvic level.

**Figure 3:**
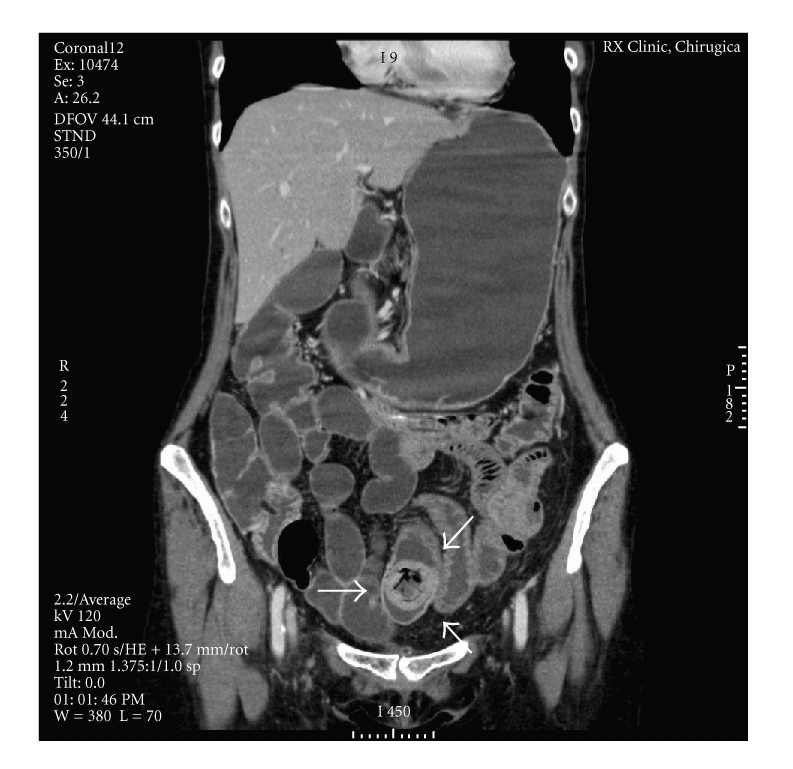
Coronal contrasted-enhanced scan shows ovoid intraluminal mass with mottled gas pattern consistent with bezoar (arrows). No dilation proximal to the site of obstruction is present.

A 60-year-old woman was admitted to our hospital with a 1-year history of recurrent epigastric and periumbilical abdominal pain with intermittent vomiting. No recent history of melena or mucus was reported. Importantly, the patient did not refer a vegetarian lifestyle, but she followed a “traditional” Mediterranean diet [3]. Her medical history included hysterectomy followed by radiotherapy 19 years before and a subsequent operation for adhesion syndrome at the jejunum level. Physical examination revealed a soft abdomen and normal bowel sounds. Laboratory findings on admission were normal with the exception of a mild reduction of the *γ*-globulins (0.46 g/dL). A plain radiography of the abdomen did not provide any diagnostic clue. She underwent an endoscopic study of the colon that was conducted up to the terminal ileum. An upper gastrointestinal endoscopy was also performed. Both studies did not demonstrate any relevant abnormalities. Computed tomography with contrast medium revealed an ovoid, nonhomogeneous, intraluminal filling defect about 3 cm in size at the pelvic level inside an ileum ansa (Figures 1  and 2). No dilation proximal to the site of obstruction was observed (Figure 3). At surgery, a 3-cm phytobezoar localized at about 60 cm from the ileocaecal valve and composed of vegetable fibers was removed with an enterotomy and resection of about 15 cm of ileum affected by a significant but not complete stenosis. Histological analysis of the mucosa revealed vascular congestion, fibrosis of several areas of the serosa and of the subserosa with an increase of thickness of the vessel walls suggestive of an inflammatory radiation-induced damage. After removal of the foreign body, the patient was asymptomatic for abdominal pain, vomiting, and constipation. She was discharged under low dietary fibre intake. After two months from surgery, she restarted a Mediterranean diet. Then, the patient was followed bimonthly by clinical examinations, and she is still asymptomatic after two-year follow-up.

## 3. Discussion

Bezoars are composed by ingested foreign materials that accumulate within the gastrointestinal tract [4]. They are typically named according to the type of the substance that makes up their composition; the five major subgroups are (1) phytobezoars; (2) pharmacobezoars (composed of undigested medications); (3) trichobezoars (composed of hair, above all in young women affected by trichotillomania); (4) lactobezoars (reported only in neonatal period and consisting of ingested milk); (5) foreign body bezoars (chewing gum, candy, toilet paper, and sunflower seeds) [1, 5, 6]. Phytobezoars represent the most frequent type of bezoar. They are composed of undigestible vegetable or fruit fibers, for example cellulose and tannin. Various kinds of fruits or vegetables could be implicated in the formation of these foreign bodies, but the most well recognized are persimmons, in which there is a high concentration of tannin that precipitate when coming into contact with gastric acid. The most common site of formation of a phytobezoar is the stomach where it often generates gastric ulcers; however, it is not unusual to find parts of phytobezoar into the small bowel, above all in the jejunum and proximal ileum, where they can become impacted and cause luminal obstruction [7–9]. Reports of bezoars causing obstruction of the gastrointestinal tract can be found dating back to the late 18th century; more recently, a metanalysis conducted upon 19 studies published between 1994 and 2005 showed that out of 996 cases of small bowel acute obstruction, 8 were due to a bezoar of the small bowel (0.8% of all the etiologies) [10]. This sets bezoar as the fifth most frequent cause of acute small bowel obstruction after adhesions (83.2%), external hernia (3.1%), malignancy (2.9%), and internal hernia (1.9%) [11]. Abdominal plain radiography may show a filling defect in the gastrointestinal tract outlined by gas [12]. For phytobezoars causing intestinal obstruction, instead, CT has become the most useful tool of diagnosis [13]. Pathognomonic signs are a well-defined ovoid intraluminal mass with mottled gas pattern at the site of obstruction, a focal transition zone with dilated fluid and air-filled loops of the small bowel proximal site and collapsed loops of small bowel distal to the site of obstruction [14]. To date, no standardized treatment method exists. The most common method for bezoar removal is chemical dissolution, surgical removal, and endoscopic removal. In this case, surgical treatment was the only choice as the phytobezoar was symptomatic for intestinal obstruction [1, 15]. The main risk factors for bezoar formation are poor mastication, vegetarian diet, diabetic gastroparesis, vagotomy, and previous gastric surgery, such as Billroth I or II operations as well as all the conditions of impaired gastric motility [16–18]. In a recent study enrolling 18 subjects, Zamir et al. showed that 44% patients had no history of gastric surgery but they had other probable predisposing factors, such as haemodialysis and narcotic or anticholinergic use [19, 20]. It is possible that other, not yet well defined, conditions could be implicated in phytobezoar aetiology and pathogenesis. To our knowledge, this is a rare case of a small bowel phytobezoar correlated to radiation-induced terminal ileal stricture. Recently Wilson and coworkers also documented a case of a woman affected by small bowel obstruction after a hysterectomy followed by radiotherapy twenty years before [21]. No other well-known risk factor was observed, including evidence of any adhesion. Similarly, in the present case report, no conventional condition related to a bezoar formation was present. These two cases mainly differentiate for the duration of abdominal pain and constipation. In the present case, the patient presented a long history of abdominal pain and constipation (1 year), whereas in the other report the pain lasted only 2 weeks. Therefore, these reports suggest that phytobezoar might be a possible cause of both chronic intestinal subocclusion and acute intestinal occlusion in patients with previous radiotherapy and subsequent ileal stenosis and altered gastrointestinal motility.

## 4. Conclusions

Bezoars consist of ingested foreign materials that accumulate within the gastrointestinal tract. Phytobezoars represent the most frequent type of bezoar and they can cause intestinal obstruction. Computed tomography has become the most useful tool of diagnosis, as in the case here reported. The best known risk factors are all the situations of impaired gastric motility. In the case that is shown, no conventional factor can be recognized. It is possible that other, not yet identified, predisposing conditions exist such as a radiation-induced altered peristalsis or small bowel stenosis. In the case of clinical presentation of intestinal subocclusion bezoar must be considered as a potential, although rare, tractable cause.
